# Primary ovarian endometrial stromal sarcoma after hysterectomy associated with multiple organ invasion: A case report

**DOI:** 10.1097/MD.0000000000033306

**Published:** 2023-03-24

**Authors:** Ningxin Chen, Min Gong, Wen Lai, Ling Ling, Qiaoling Liu

**Affiliations:** a Department of Obstetrics and Gynecology, Nanjing Medical University, Nanjing, Jiangsu, China; b Department of Obstetrics and Gynecology, The Affiliated Jiangning Hospital with Nanjing Medical University, Nanjing, Jiangsu, China.

**Keywords:** endometrial stromal sarcoma, extra-uterine, invasion, low-grade, ovary

## Abstract

Endometrial stromal sarcoma (ESS) is a rare disease in patients with uterine malignancies, accounting for <1%. Low-grade endometrial stromal sarcoma (LGESS) accounts merely 0.2% of gynecologic malignant tumor. Primary low-grade extrauterine endometrioid stromal sarcomas (LGEESS) is even more uncommon, with only a few documented case reports. We report a case of primary LGEESS exhibiting widely invasion in multiple organs after hysterectomy, which is the first case reported in Jiangsu Province of China.

**Patient concerns:**

A 42-year-old nulliparous female with dysgnosia presented with a moderate amount of irregular vaginal bleeding, abdominal pain and distension, and frequent urination for 2 days. Her surgical history included a total hysterectomy and bilateral salpingectomy for uterine fibroids 6 years ago. Ultrasonography and the abdominal and pelvic computed tomography scan detected some solid polycystic masses in the pelvic and abdominal cavities.

**Diagnoses:**

The histopathology of the specimen confirmed the diagnosis of LESS in the absence of florid endometriosis. The patient was diagnosed with primary extrauterine endometrial stromal sarcoma at FIGO stage III.

**Interventions:**

Surgery and histopathology were performed.

**Outcome:**

After surgery, the patient was maintained on leuprorelin acetate microspheres with sustained release for injection at 3.75 mg once every 4 weeks while refusing further radiotherapy.

**Lessons:**

The diagnosis of primary LGEESS is challenging mainly because of their unforeseen location and nongynecologic signs and symptoms. Total hysterectomy and bilateral salpingo-oophorectomy are recommended to LGESS, while additional resection for extrauterine disease depends on disease extent and resectability.

## 1. Introduction

Endometrial stromal sarcoma (ESS) is a rare disease in patients with uterine malignancies accounting for <1%. 2020 WHO classification schema has identified ESS as low-grade endometrial stromal sarcoma (LGESS) and high-grade ESS.^[1]^ LGESS accounts for merely 0.2% of the gynecologic malignant tumor. It is even more uncommon for primary low-grade extrauterine endometrioid stromal sarcomas (LGEESS) because it develops at extrauterine sites especially including the peritoneal surfaces, intestinal wall, ovaries, pelvis, vagina, urinary bladder, and fallopian tubes, without involving uterine.^[2,3]^

Here, we report a case of primary LGEESS exhibiting wide invasion in multiple organs after hysterectomy, which is the first case reported in Jiangsu Province of China.

## 2. Case presentation

A 42-year-old nulliparous female with a diagnosis presented with a moderate amount of irregular vaginal bleeding, abdominal pain and distension, and frequent urination for 2 days. Her surgical history included a total hysterectomy and bilateral salpingectomy for uterine fibroids 6 years ago. Per vaginal examination revealed a 15-mm-diameter red lesion on the vaginal stump. On gynecological examination, a 10-cm-diameter nonmobile lump was palpable with tenderness over the right adnexa.

**Figure 1.**
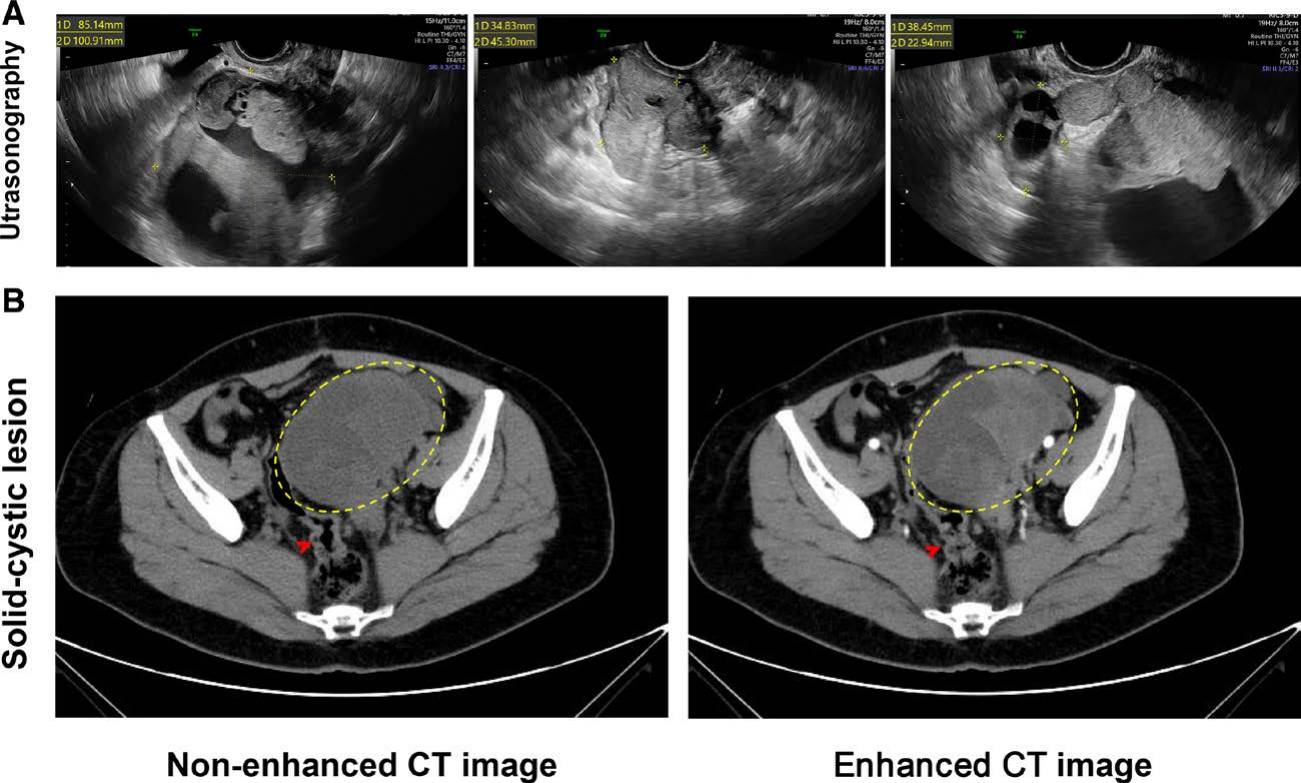
(a) Ultrasonography detected some solid polycystic masses in the pelvic and abdominal cavities (85.1 × 100.9 × 87.4 mm heterogeneous lesion in the pelvis and abdominal cavity, 38.4 × 22.9 × 39.8 mm oval echogenicity in the right pelvis, and 34.8 × 45.3 mm dense echogenicity in the backward of the pelvis). (b) An abdominal and pelvic computed tomography scan demonstrated a solid-cystic lesion of 102 × 96 × 103 mm in the left pelvis, speculated to originate from the left ovary and behaved similarly to ovarian cystic adenocarcinoma. The red arrow points to the thickened sigmoid colon.

Ultrasonography detected some solid polycystic masses in the pelvic and abdominal cavities (85.1 × 100.9 × 87.4 mm heterogeneous lesion in the pelvis and abdominal cavity, 38.4 × 22.9 × 39.8 mm oval echogenicity in the right pelvis, and 34.8 × 45.3 mm dense echogenicity in the backward of the pelvis) (Fig. 1a). Pelvic and abdominal effusions were also revealed. An abdominal and pelvic computed tomography scan demonstrated a solid-cystic lesion of 102 × 96 × 103 mm in the left pelvis (Fig. 1b), speculated to originate from the left ovary and behaved similarly to ovarian cystic adenocarcinoma. Multiple soft tissue nodules showed contrast enhancement in the pelvis and lower right abdomen with sigmoidocolic thickening (Fig. 1b), suggesting dissemination and sigmoidocolic local invasion. A 23 × 29 mm well-defined circumscribed lesion in the right pelvic cavity was cystic in appearance.

The CA125 level showed a significant increase to 367.00 U/mL, and CA19–9 was increased to 29.00 U/mL.

As discussed in a multidisciplinary tumor board, the patient was planned for surgery.

Intraoperative findings revealed a 2 cm off-white lump in the anterior wall of the sigmoid colon with multiple small metastases at the ileocolic mesentery. Laparotomy revealed a solid left cystic adnexal mass as well as a bladder adherent to the intestine. The 56 cm hard, nonmobile lesion was palpable in the left pelvis, with a lower boundary that reached the left paratrovaginalis and invaded the full layer of the left vaginal apex. The left side reached the left pelvic wall. The left internal iliac vessels, as well as the lower segment of the left ureter, passed through the tumor, and the boundary of the tumor was unclear. The lesion was compressing the left ureter and causing the left hydroureter. the boundary between the pelvic floor mass and the rectum was unclear.

The patient underwent a transabdominal pelvic adhesiolysis, bilateral oophorectomy, limited resection of the pelvic floor lesion, and tumor resection of the anterior wall of the sigmoid flexure with a sigmoid colostomy.

**Figure 2.**
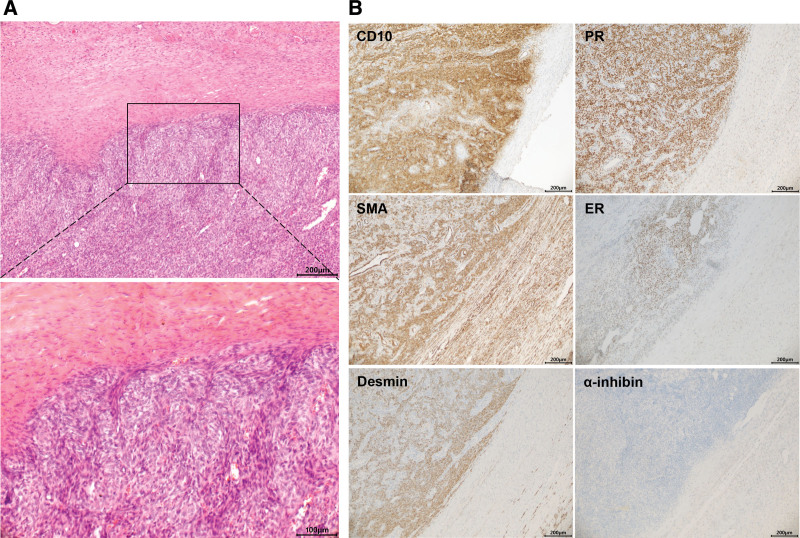
(a) HE staining result of the intraoperative specimen revealed features of LGEESS with spindle-shaped atypia and invasive growth cells. (b) The tumor cells were performed with immunohistochemical staining and showed a diffusely positive for CD 10, desmin, smooth muscle actin, estrogen, and progesterone receptors (and 10% positive for Ki-67), but negative for α-inhibin. LGEESS = low-grade extrauterine endometrioid stromal sarcomas.

HE staining result of the intraoperative specimen revealed features of LGEESS with spindle-shaped atypia and invasive growth cells (Fig. 2a). The tumor cells were performed with immunohistochemical staining and showed a diffusely positive for CD 10, desmin, smooth muscle actin, estrogen, and progesterone receptors (and 10% positive for Ki-67), but negative for α-inhibin, indicating features of LGESS (Fig. 2b). The pelvis and sigmoid flexure showed infiltration by LGESS. Over the rectum, the left ureter, the mesocolon, and the vagino also showed tumor deposits.

After surgery, she was maintained on leuprorelin acetate microspheres with sustained release for injection at 3.75 mg once every 4 weeks while refusing further radiotherapy.

## 3. Discussion

The occurrence of ESS in extrauterine locales is exceedingly rare, particularly in the absence of metastasis or extension of a primary neoplasm. The natural history of LGESS is that of an indolent, slow-growing tumor.^[4]^ There are still a few patients who may initially present with advanced tumors, though the majority of patients, with disease confined to the uterus, leading to a favorable prognosis.^[5]^ In our case, the patient was diagnosed with primary extrauterine ESS at The International Federation of Gynecology and Obstetrics stage III.^[5]^

The diagnosis of primary LGEESS is challenging mainly because of its unforeseen location and nongynecologic signs and symptoms. The clinician needs to eliminate sex cord-stromal tumors, adult granulosa cell tumors, fibroma, thecoma, and fibrosarcoma before confirming that the uterus is free of lesions.^[3]^

Total hysterectomy and bilateral salpingo-oophorectomy are recommended to LGESS, while additional resection for extrauterine disease depends on disease extent and resectability.^[6,7]^ The efficacy of adjuvant antiestrogen hormone therapy to reduce the risk of recurrence has wide Acknowledgments because estrogen receptors and progesterone receptors are highly expressed in LGESS. Hence adjuvant hormone therapy is recommended for stage II to IV LGESS.^[6]^ The treatment guidelines of LGEESS are the same as LGESS.^[3]^

LGESS rarely occurs at extra-uterine sites (either as primary or secondary lesions). However, when they do, they pose challenges in diagnosis. Extra-uterine manifestation and histological features, in conjunction with immunohistochemical and molecular findings, are essential for making an accurate diagnosis. Although extremely rare, treatment guidelines of extra-uterine LGESS are based on those for uterine ESS. Advance trials with a larger number of patients, longer follow-up times, and uniform treatment modalities are needed to clarify the roles of chemotherapy, hormonal therapy, and radiotherapy in LGESS.

## Author contributions

**Investigation:** Ningxin Chen.

**Project administration:** Min Gong, Qiaoling Liu.

**Resources:** Qiaoling Liu.

**Supervision:** Min Gong, Qiaoling Liu.

**Writing – original draft:** Ningxin Chen, Min Gong, Wen Lai.

**Writing – review & editing:** Ling Ling, Qiaoling Liu.
